# Human Papillomaviruses Activate the ATM DNA Damage Pathway for Viral Genome Amplification upon Differentiation

**DOI:** 10.1371/journal.ppat.1000605

**Published:** 2009-10-02

**Authors:** Cary A. Moody, Laimonis A. Laimins

**Affiliations:** Department of Microbiology and Immunology, Feinberg School of Medicine, Northwestern University, Chicago, Illinois, United States of America; Fred Hutchinson Cancer Research Center, United States of America

## Abstract

Human papillomaviruses (HPV) are the causative agents of cervical cancers. The infectious HPV life cycle is closely linked to the differentiation state of the host epithelia, with viral genome amplification, late gene expression and virion production restricted to suprabasal cells. The E6 and E7 proteins provide an environment conducive to DNA synthesis upon differentiation, but little is known concerning the mechanisms that regulate productive viral genome amplification. Using keratinocytes that stably maintain HPV-31 episomes, and chemical inhibitors, we demonstrate that viral proteins activate the ATM DNA damage response in differentiating cells, as indicated by phosphorylation of CHK2, BRCA1 and NBS1. This activation is necessary for viral genome amplification, as well as for formation of viral replication foci. In contrast, inhibition of ATM kinase activity in undifferentiated keratinocytes had no effect on the stable maintenance of viral genomes. Previous studies have shown that HPVs induce low levels of caspase 3/7 activation upon differentiation and that this is important for cleavage of the E1 replication protein and genome amplification. Our studies demonstrate that caspase cleavage is induced upon differentiation of HPV positive cells through the action of the DNA damage protein kinase CHK2, which may be activated as a result of E7 binding to the ATM kinase. These findings identify a major regulatory mechanism responsible for productive HPV replication in differentiating cells. Our results have potential implications for the development of anti-viral therapies to treat HPV infections.

## Author Summary

Over 100 types of human papillomavirus (HPV) have been identified, and approximately one-third of these infect epithelial cells of the genital mucosa. A subset of these HPV types are the causative agents of cervical and other anogenital cancers. The infectious life cycle of HPV is dependent on differentiation of the host epithelial cell, with viral genome amplification and virion production restricted to differentiated suprabasal cells. While normal keratinocytes exit the cell cycle upon differentiation, HPV positive suprabasal cells are able to re-enter S-phase to mediate productive replication. The mechanisms regulating the activation of differentiation-dependent viral replication are largely unknown. In this study, we demonstrate that HPV induces an ATM-dependent DNA damage response that is essential for viral genome amplification in differentiating cells. In addition, we have found that ATM signaling to its downstream target CHK2 is critical for providing an environment that is conducive to HPV productive replication. Our findings identify an important regulatory mechanism by which HPV controls replication during the productive phase of the life cycle and may identify new targets for the development of therapeutics to treat HPV-induced infections.

## Introduction

Human papillomaviruses (HPV) are the etiological agents of most anogenital cancers and their productive life cycle is dependent upon epithelial differentiation [1],[2]. HPVs infect cells in the basal layer of stratified epithelia, but restrict the productive phase of the life cycle to highly differentiated suprabasal cells [3]. Viral genome amplification, late gene expression and virion production are induced in suprabasal cells that have re-entered S-phase. In undifferentiated basal cells, viral genomes are maintained as episomes at approximately 100 copies per cell and replicate in synchrony with cellular replication. In contrast, upon differentiation HPV genomes are replicated to thousands of copies per cell in a process referred to as amplification [4]. While normal epithelial cells exit the cell cycle upon differentiation, HPV-infected cells are able to over-ride normal checkpoint controls and remain active in the cell cycle, allowing for the synthesis of cellular proteins that are necessary for viral replication [5],[6]. HPV proteins activate low levels of caspases belonging to the intrinsic pathway in differentiating cells, and this is necessary for viral replication [7]. The mechanisms regulating productive replication of HPVs upon differentiation, however, remain largely unknown.

The fidelity of cellular replication is controlled by signaling pathways that block the propagation of damaged DNA [8],[9]. Central to these repair pathways are the ATM (ataxia-telangiectasia mutated), and ATR (ATM and Rad3-related) kinases, which belong to a structurally related family of serine-threonine kinases that share a PI-3 kinase-like domain, but only phosphorylate proteins [9]. ATM is a prime mediator of the cellular response to double strand breaks [10], while ATR controls the response to UV damage, as well as stalled DNA replication forks [11]. Both kinases act in part by controlling cell cycle checkpoints at G1, S and G2. Several viruses have been shown to interact with and/or affect components of the ATM DNA damage pathway [12]. Herpes simplex virus (HSV) induces an ATM-damage response as soon as pre-replication centers are formed, and this activation is essential for productive replication [13],[14]. In contrast, adenovirus must mislocalize and degrade DNA repair proteins to ensure viral replication [15]. Using recombinant adenoviruses, high-level expression of HPV-16 E7 in fibroblasts was shown to activate the ATM pathway [16], but it is unclear whether these effects are physiologically significant, or if they play any role in the viral life cycle.

ATM activates a number of downstream targets that are involved in cell cycle control, apoptotic responses and DNA repair [17]. These proteins can be divided into three pathways that lead to activation of cell cycle checkpoints: a p53/mdm2 pathway, a CHK2 branch, and a NBS1/BRCA1/SMC1 pathway. ATM directly activates p53 by phosphorylation at serine 15, as well as by phosphorylating Mdm2, the ubiquitin ligase that regulates p53 stability [18],[19],[20]. In the second pathway, ATM phosphorylates CHK2 leading to arrest in S- and G2-phases by inhibiting the action of Cdc25 phosphatases [21],[22]. Another branch of S-phase checkpoint control involves ATM targeting of NBS1, a member of the MRN double strand break repair complex [23]; BRCA1, the breast cancer susceptibility protein [24]; and SMC1, a cohesin binding protein [25],[26]. An additional downstream activity of ATM, as well as ATR, that is important for S-phase checkpoint control is phosphorylation of the tail of a histone variant H2AX (γ-H2AX), which leads to recruitment of DNA damage regulatory factors to distinct nuclear foci [27],[28]. Phosphorylation of these, as well as other targets allows for DNA damage to be assessed and for repair to take place.

Given the importance of this pathway in controlling replication, we investigated if ATM signaling was necessary for stable HPV replication in undifferentiated cells, as well as productive replication in differentiated cells. Our studies indicate that HPV proteins induce an ATM response in both undifferentiated and differentiated cells. Importantly, we found that ATM kinase activity is necessary for viral genome amplification in differentiating cells, but not for stable maintenance in undifferentiated cells. These studies implicate HPV activation of DNA damage signaling in controlling productive viral replication upon differentiation.

## Results

### HPV induces a DNA damage response

**Figure 1:**
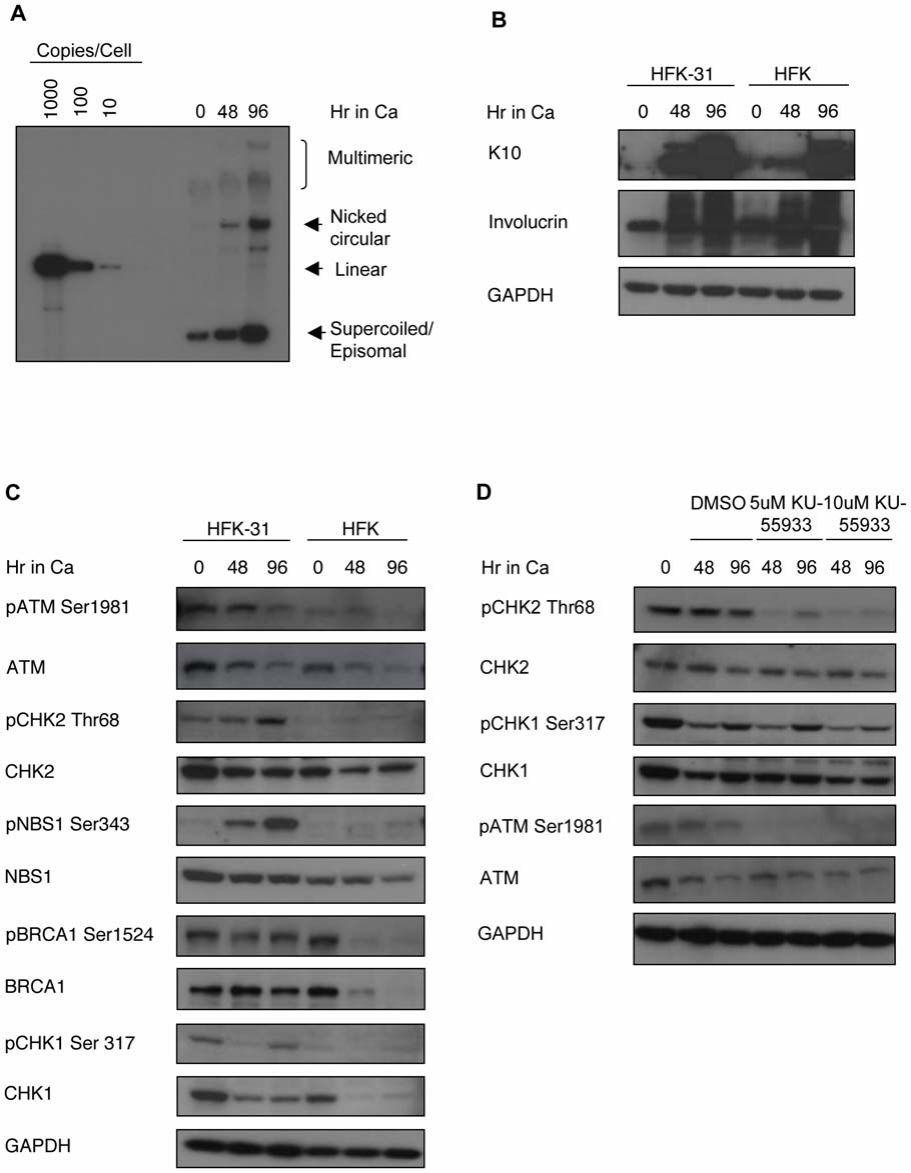
HPV induces a cellular DNA damage response in infected cells. (A) DNA was harvested from undifferentiated (T0) HPV positive cells, as well as after 48 and 96 hours of differentiation in high calcium. Southern blot analysis was performed to examine amplification of viral genomes, represented by supercoiled (episomal) viral DNA. Standards of copies of HPV genomes per cell are indicated to the left of the gel. (B) Lysates harvested from undifferentiated (T0) HPV-31 positive HFKs (HFK-31) and from normal HFKs, as well as cells differentiated in high calcium medium for 48 and 96 hours, were analyzed by Western blot analysis for levels of the differentiation markers keratin 10 (K10) and involucrin. (C) Lysates were harvested from undifferentiated HFK-31 and from normal HFKs, as well as cells differentiated in high calcium medium for 48 and 96 hours. Western blot analysis was performed using the indicated phospho-specific antibodies, as well as antibodies to detect total protein levels. (D) HFK-31 cells were treated with DMSO as a vehicle control, or the indicated amounts of the ATM inhibitor KU-55933 for 48 and 96 in high calcium medium. Lysates were harvested at the indicated times and analyzed by Western blot analysis for the activation of CHK2 (Thr68), CHK1 (Ser317), and ATM (Ser1981), as well as antibodies to detect total protein levels. GAPDH served as a loading control. Ca = calcium.

To determine if HPV induces a DNA damage response in infected cells we first examined the expression level, as well as activation status, of ATM by Western blot analysis (Figure 1C). For these studies, we examined undifferentiated, as well as differentiated human keratinocyte cell lines that maintain HPV-31 episomes at approximately 50 copies per cell, and compared effects to a matched set of normal human keratinocytes isolated from foreskin circumcisions (HFKs). We have shown previously that calcium-induced differentiation is sufficient to activate the productive phase of the HPV life cycle by 48 hours [7]. Upon differentiation, approximately 25% of HPV positive cells re-enter S phase and undergo viral genome amplification, resulting in high levels of episomal DNA [29] (Figure 1A). The induction of differentiation is indicated by the expression of cellular proteins, such as keratin 10 (K10) and involucrin (Figure 1B). As shown in Figure 1C, the total levels of ATM were similar between HPV-31 positive HFKs (HFK-31) and matched normal HFKs in undifferentiated cultures, as well as after 48 and 96 hours of differentiation in high calcium medium. In response to double-strand breaks or changes in chromatin, ATM is activated through the autophosphorylation of inactive, dimeric ATM on serine 1981, followed by dissociation into active monomers [30]. The phosphorylated form of ATM (Ser1981) was detected in undifferentiated HPV-31 positive cells and maintained at a similar level through 48 hours of differentiation, with a decrease occurring at 96 hours (Figure 1C). In contrast, only a low level of pATM Ser1981 was observed in normal HFKs. This pattern of ATM phosphorylation was observed in multiple independently derived polyclonal populations of HFK-31 cells, and matched normal HFKs, as well as in CIN612 cells, which is a clonal cell line derived from a HPV-31 positive biopsy (Bedell MA, 1991) (data not shown).

To determine if phosphorylation of ATM correlated with activation of downstream targets, we examined the phosphorylation status of three of its substrates: CHK2, NBS1 and BRCA1. CHK2 is an important transducer of the ATM signaling pathway, and its activation is initiated by ATM phosphorylation on threonine 68 (pCHK2) [21],[22]. While pCHK2 was detected in both undifferentiated and differentiated HPV-31 positive cells, only low levels were detected in normal HFKs (Figure 1C), which correlates with the low level of pATM detected in these cells. Activation of the ATM pathway can also result in the phosphorylation of NBS1 and BRCA1, both of which play important roles in DNA repair and the regulation of S and G2 checkpoints [24],[31],[32],[33]. In HFK-31 cells, BRCA1 was phosphorylated at all states of differentiation (Figure 1C). While we also observed pBRCA1 in undifferentiated normal HFKs, the levels rapidly declined along with total BRCA1 upon differentiation. Phosphorylation of BRCA1 in normal HFKs may be due to either low level activation of ATM, or through the action of ATR, which can also phosphorylate BRCA1 [24]. Importantly, phosphorylation of NBS1 at Ser343 was induced only upon differentiation of HPV positive cells, with low levels observed in undifferentiated cells (Figure 1C). In contrast, little phosphorylation of NBS1 was observed in either undifferentiated or differentiated normal HFKs (Figure 1C). These results indicate that phosphorylation of NBS1 in HPV positive cells is differentiation-specific, and correlates with the induction of productive viral replication (Figure 1A). Interestingly, we observed phosphorylation of the ATR substrate CHK1 on Ser317 in undifferentiated HPV positive cells (Figure 1C), suggesting that ATR is also active. However, the levels of phosphorylated CHK1, as well as total CHK1, decreased by 48 hours post-differentiation. Similar effects were observed in multiple independently derived HFK-31 cell lines, as well as in CIN612 cells (data not shown). Overall, these results indicate that HPV proteins induce a DNA damage response that is maintained throughout the viral life cycle and characterized by the activation of the ATM substrates CHK2, NBS1 and BRCA1.

We next wanted to confirm that ATM activity was responsible for CHK2 phosphorylation in HPV positive cells, as CHK2 can also be phosphorylated and activated by the ATR kinase [22],[34]. HFK-31 cells were treated with a small molecule inhibitor of ATM, KU-55933, that inhibits kinase activity without affecting the total levels of ATM [35] (Figure 1D). Treatment of HFK-31 cells with 5 or 10 uM of the ATM inhibitor resulted in significantly reduced phosphorylation of CHK2, as well as ATM itself in differentiated cells (Figure 1D). Treatment of HPV-31 cells with this inhibitor also resulted in decreased phosphorylation of NBS1 and BRCA1 (data not shown), while phosphorylation of CHK1 Ser317 was only minimally affected with 10 uM of the inhibitor (Figure 1D). Treatment of undifferentiated HPV-31 positive cells also resulted in inhibition of CHK2 phosphorylation without affecting CHK1 phosphorylation (data not shown). These results indicate that ATM activity is necessary for CHK2 phosphorylation in cells maintaining HPV genomes. In addition, our findings indicate that the low level of CHK1 phosphorylation observed in differentiating HPV positive cells is ATM-independent, and may suggest a role for ATR in the non-productive phase of the life cycle.

### HPV does not induce degradation of MRN components

**Figure 2:**
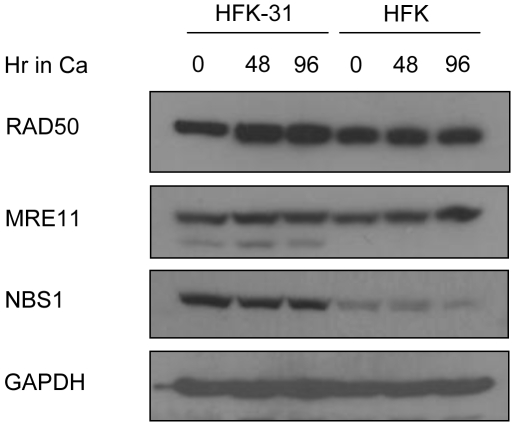
HPV does not affect the levels of the components of the MRN complex. Lysates were harvested from undifferentiated HFK-31 cells and normal HFKs, as well as from cells induced to differentiate in high calcium for 48 and 96 hours. Western blot analysis was performed using antibodies to NBS1, MRE11 and RAD50. Western blots were stripped and re-probed with an antibody to GAPDH as a loading control. Ca = calcium.

In response to DNA damage, ATM is recruited to distinct nuclear foci by the MRN complex, which consists of NBS1, MRE11 and RAD50 [36],[37]. Recruitment to double strand breaks allows for ATM-dependent phosphorylation of at least a subset of downstream targets [10],[38]. Adenoviruses abrogate the ATM response by relocalizing and degrading components of the MRN complex, which would normally promote the detrimental concatemerization of viral genomes [15],[39]. For HPV, the amounts of MRE11 and RAD50 were found to be similar in both undifferentiated and differentiated cells, as well as in HFKs, while the levels of NBS1 were consistently higher in HPV-31 cells compared to normal HFKs (Figure 2). In addition, components of the MRN complex were localized to nuclear foci in HPV positive cells (Figure S1). Overall, these results indicate that HPV does not induce degradation of these repair proteins in order to facilitate the viral life cycle, but instead maintains them at high levels throughout differentiation.

### HPV induces the accumulation of activated ATM, CHK2 and γ-H2AX in nuclear foci

**Figure 3:**
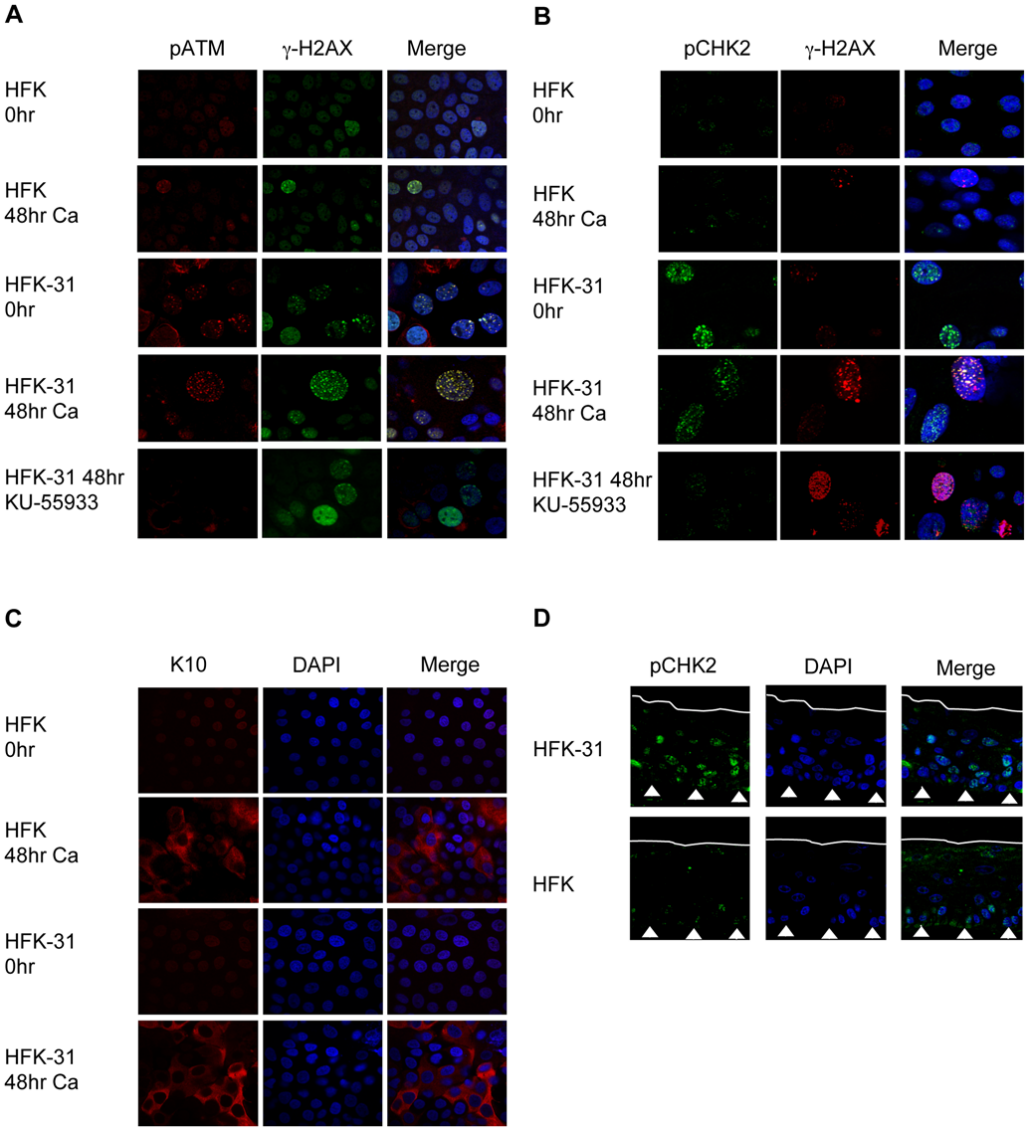
HPV induces ATM activation and the accumulation of DNA repair proteins in nuclear foci. HFK-31 cells, as well as normal HFKs were harvested, fixed and permeabilized at either time 0 (undifferentiated cells), or after 48 hours of differentiation in high calcium medium containing DMSO, or 10 uM of the ATM inhibitor KU-55933. Samples were stained with (A) anti-pATM Ser1981 (red), anti-H2AX Ser139 (γ-H2AX) (green) antibodies; (B) anti-pCHK2 Thr68 (green), anti-γ-H2AX (red) antibodies; or (C) an anti-K10 antibody as a marker of differentiation. Samples were subsequently analyzed by confocal laser scanning microscopy using either a 60× objective lens (panels A and B), or a 40× objective lens (panel C). Cellular DNA was counterstained with DAPI. (D) Sections from organotypic raft cultures generated from HFK-31 cells, or normal HFKs were stained with an antibody to detect pCHK2-Thr68 (green). Cellular DNA was counterstained with DAPI. Images were visualized using confocal laser scanning microscopy. Ca = calcium.

The detection of phosphorylated CHK2, NBS1 and BRCA1 in HPV positive cells suggested that ATM may be localized to nuclear foci, as is observed in cells undergoing a DNA damage response [8]. To investigate this possibility, we examined the localization of the phosphorylated form of ATM (Ser1981) in HPV-31 positive cells by confocal fluorescence microscopy. As shown in Figure 3A, pATM Ser1981 was found in distinct nuclear foci in undifferentiated HPV-31 positive cells. pATM Ser1981 colocalized with γ-H2AX, a modified histone associated with double-stranded breaks [40], and these foci were retained upon differentiation in a similar number of HPV-31 positive cells (39.7±6.6%). In contrast, normal HFKs exhibited diffuse staining of pATM, with only a few foci being detected in both undifferentiated and differentiated cells (3.2%±.8 and 3.1±.6%, respectively). Inhibition of ATM activity by treatment with KU-55933 abrogated formation of pATM-Ser1981 foci in HPV-31 positive cells (Figure 3A), and correlates with the decrease in pATM levels observed in the presence of the inhibitor by Western blot analysis (Figure 1D). Interestingly, the number of HPV-31 positive cells that exhibited γ-H2AX foci upon 48 hours of differentiation was reduced but not completely inhibited in the presence of KU-55933 (40.7% to 10.8%), indicating that other kinases, such as ATR [27], may contribute to this activity in HPV positive cells. Similar results were observed in multiple HFK-31 lines, as well as CIN612 cells. These results suggest that HPV induces the activation and accumulation of ATM and γ-H2AX at distinct sites in the nucleus.

We next examined the localization of phosphorylated CHK2 Thr68 in undifferentiated and differentiated cells. In undifferentiated HPV-31 positive cells, a number of foci containing both pCHK2 Thr68 and γ-H2AX were detected, although every cell did not stain positive for these foci (Figure 3B). Upon differentiation in high calcium medium for 48 hours, the number of foci containing both pCHK2 and γ-H2AX was retained (21.5±1.1%). In contrast, only smaller, less intense foci containing pCHK2 and γ-H2AX were detected in undifferentiated HFKs (3.3±0.5%), and the number did not increase upon differentiation. When HPV-31 positive cells were treated with the ATM inhibitor, the formation of the pCHK2 foci was substantially diminished (21.5% to 3.4%), further supporting a dependence on ATM activity for CHK2 activation (Figure 3B). Calcium-induced differentiation of HPV-31 positive cells and normal HFKs was confirmed by staining for K10 (Figure 3C).

It was next important to confirm that pCHK2 Thr68 localization to nuclear foci was not specific to calcium-induced differentiation. For these studies, HPV-31 positive keratinocytes, as well as normal keratinocytes, were induced to differentiate by growth in organotypic raft cultures, and immunohistochemistry was performed on cross sections of the rafts. As shown in Figure 3D, pCHK2 Thr68 was detected in the basal layer of HPV-31 positive rafts, as well as in a large number of suprabasal cells (67%), while only a few cells in the basal layer of normal HFK rafts had a comparable signal (5%). These results correlate with the CHK2 activation and nuclear localization observed upon calcium-induced differentiation. In addition to CHK2, we also observed similar staining patterns for pATM Ser1981, γ-H2AX and MRE11 in rafts of HPV-31 positive cells (Figure S2). Differentiation of HFK-31 cells, as well as normal HFKs, using the raft system was confirmed by staining for K10 (Figure S2). Overall, these results indicate that an ATM DNA damage response is activated in HPV-positive cells.

### ATM and CHK2 kinase activity is required for viral genome amplification in differentiating cells

**Figure 4:**
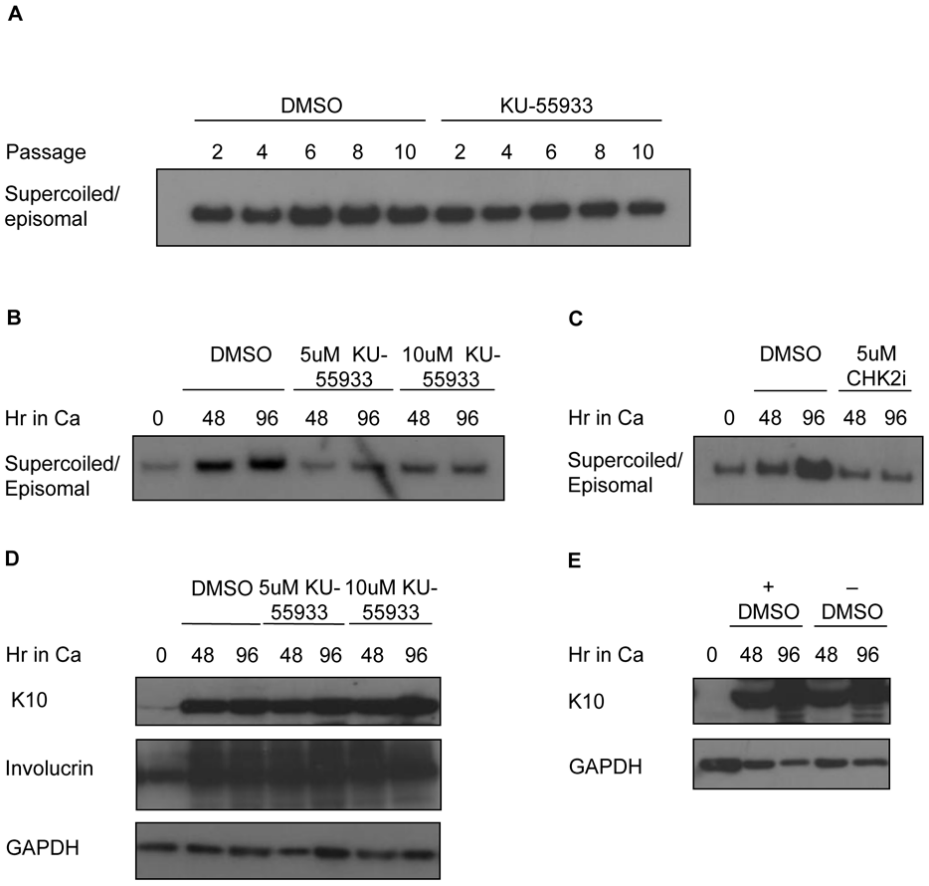
ATM activity is required for differentiation-dependent viral genome amplification, but not episomal maintenance. (A) DNA was isolated at the indicated passages from undifferentiated HPV-31 positive CIN612 cells grown in the presence of DMSO, or the ATM inhibitor KU-55933 and analyzed by Southern blot analysis for the presence of episomal (supercoiled) HPV DNA. (B, C) DNA was harvested from undifferentiated (0 hour) CIN612 cells, as well as cells differentiated in high calcium (48 and 96 hr) in the presence of DMSO, (B) 5 or 10 uM KU-55933, or (C) 5 uM CHK2 inhibitor (CHK2i) and analyzed for amplification of viral genomes by Southern blot analysis. (D) Lysates were harvested from undifferentiated (0 hour) CIN612 cells, as well as from CIN612 cells induced to differentiate in high calcium in the presence or absence of 5 or 10 uM KU-55933. Western blot analysis was performed using an antibody to keratin 10 (K10) and involucrin. (E) Lysates were harvested from undifferentiated (T0) HPV-31 positive cells, as well as from cells induced to differentiate in high calcium for 48 and 96 hours in the presence or absence of DMSO. Western blot analysis was performed using an antibody to K10. Blots were stripped and probed for GAPDH as a loading control. Ca = calcium.

We next investigated if activation of the ATM pathway is necessary for stable replication of HPV genomes in undifferentiated cells, or viral genome amplification in differentiated cells. Since our studies identified activated CHK2 and BRCA1 in undifferentiated HPV-positive cells, we first examined the effect of inhibiting ATM kinase activity on episomal maintenance (Figure 4A). HPV-31 positive cells were grown in monolayer cultures and treated every two days with either DMSO as a vehicle control, or 5 uM KU-55933 to inhibit ATM kinase activity. The cells were passaged every five days and DNA was harvested at every other passage. Southern blot analysis was then performed to examine the status of the episomal viral DNA. As shown in Figure 4A, cells treated with DMSO maintained a similar number of episomes over time. We also found that cells treated with the ATM inhibitor exhibited only modest fluctuations in copy number as a function of extended passage. This experiment was performed multiple times with similar results. Since viral episomes were not rapidly lost upon passaging, these results indicate that ATM activity is not essential for the stable maintenance of episomes in undifferentiated cells.

To determine if ATM kinase activity is necessary for differentiation-dependent viral genome amplification, HPV-31 positive CIN612 cells were induced to differentiate in high calcium medium in the presence of DMSO or KU-55933. DNA was harvested from monolayer cells (0 hour), as well as 48 and 96 hours post-differentiation. As shown in Figure 4B, Southern blot analysis for HPV DNA demonstrated that cells treated with the ATM inhibitor exhibited significantly impaired viral genome amplification as compared to cells treated with DMSO alone. This experiment was repeated five times with identical results, demonstrating that ATM activity is necessary for productive viral replication. Since CHK2 is a major transducer of ATM signaling, we next wanted to determine if CHK2 activity is necessary for viral replication in differentiating cells. For this study, we used a specific inhibitor of CHK2 activity that effectively blocks phosphorylation of downstream targets, without affecting its own phosphorylation (Figure S4). Similarly to the ATM inhibitor, treatment of HPV-31 positive cells with the CHK2 inhibitor resulted in greatly diminished viral genome amplification upon differentiation (Figure 4C), indicating that ATM signaling to CHK2 is essential for this activity. Similar effects were also seen upon treatment of HFK-31 cell lines with the ATM and CHK2 inhibitors (data not shown), as well as when viral DNA was linearized by restriction digestion (Figure S3). Since epithelial differentiation is necessary for activation of viral genome amplification [41], we wanted to ensure that KU-55933 treatment did not act indirectly by blocking epithelial differentiation. For this analysis, we examined the expression levels of the differentiation-specific markers K10 and involucrin by Western blot analysis (Figure 4D). The subpopulation of differentiating cells that are amplifying HPV DNA are in S or G2 phase and do not express K10, while adjoining cells in G0/G1 synthesize high levels of K10 [29]. This is consistent with studies showing that upon keratinocyte differentiation, K10 is expressed in post-mitotic cells that are still metabolically active [42]. Treatment of HPV-31 positive cells with concentrations of KU-55933 up to 10 uM minimally affected K10 and involucrin expression, indicating that ATM inhibition does not alter differentiation (Figure 4D). To ensure that DMSO does not affect epithelial differentiation, we compared K10 expression between HPV-31 positive cells induced to differentiate in high calcium in the presence or absence of DMSO, and found no differences (Figure 4E).

**Figure 5:**
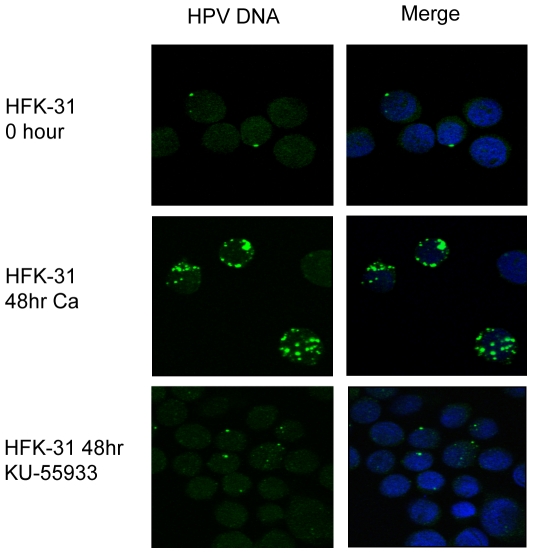
Formation of replication foci is impaired in the absence of ATM activity. FISH analysis was performed using undifferentiated (T0), as well as differentiated (48 hr high calcium) HPV-31 positive HFKs using a HPV-31 nick translated probe (green). FISH analysis was also performed on HPV positive cells grown in high calcium for 48 hr in the presence of 10 uM KU-55933. Cellular DNA was counterstained with DAPI, and images were visualized using confocal laser scanning microscopy. Ca = calcium.

Since we observed the formation of nuclear foci containing pATM Ser1981 in HPV-31 positive cells and found that ATM kinase activity is necessary for viral genome amplification, we investigated whether ATM activity is required for the formation of HPV replication foci in differentiating cells. For these studies, tyramide-enhanced fluorescence in situ hybridization (FISH) was used to detect viral genomes in cells that were treated with DMSO, or 10 uM of the ATM inhibitor. In monolayer cells, only single foci of viral genomes were detected in a small number of cells (9.6±2.3%) (Figure 5), which is consistent with previous observations [29]. In contrast, after differentiation for 48 hours in high calcium medium, the number of cells containing viral genome foci, as well as number and size of the foci, greatly increased (48±1.9%). Treatment with the ATM inhibitor prevented the formation of multiple foci per cell, resulting in a staining pattern similar to that of undifferentiated cells, providing further evidence that ATM activity is necessary for viral replication in differentiating cells.

### HPV activates CHK2 to induce caspase cleavage upon differentiation

**Figure 6:**
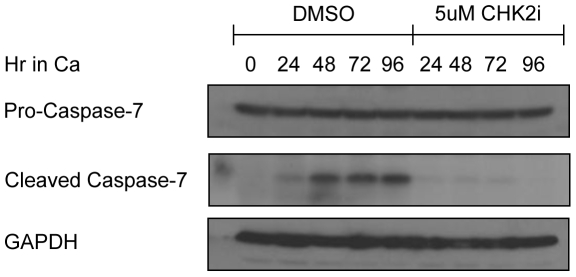
HPV-mediated caspase activation requires CHK2 kinase activity. Lysates were harvested from undifferentiated (0 hour) HPV-31 positive CIN612 cells, as well as from HPV-31 cells induced to differentiate in high calcium in the presence of DMSO or 5 uM of the CHK2 inhibitor (CHK2i) for the indicated times. Western blot analysis was performed using antibodies to detect the cleaved (active) form of caspase-7, as well as an antibody to detect pro-caspase-7. GAPDH served as a loading control. Ca = calcium.

We previously showed that HPVs induce low-levels of caspase 3/7 activation upon differentiation and that this is important for cleavage of the E1 replication protein and genome amplification [7]. The studies described above indicate that HPV requires CHK2 activity for productive replication (Figure 4C). In addition to its activity in DNA repair and cell cycle checkpoint function, CHK2 also plays a role in damage-induced apoptosis [16],[43],[44],[45], and thus could potentially contribute to the caspase activation observed in differentiating HPV-31 positive cells. To investigate this possibility, HPV-31 CIN612 cells were induced to differentiate in high calcium in the presence of DMSO or 5 uM of the CHK2 inhibitor. Cells extracts were harvested as a function of time and examined by Western blot analysis for cleavage of caspase-7. As shown in Figure 6, inhibition of CHK2 significantly impaired caspase-7 cleavage as compared to cells treated with DMSO alone, without affecting total levels of caspase-7. Similar results were found upon treatment of HFK-31 cells with the CHK2 inhibitor (data not shown). This finding, coupled with the observation that CHK2 is required for productive replication, indicates that HPVs utilize ATM signaling to promote caspase activation through CHK2, allowing for enhanced viral replication in differentiating cells.

### High-risk E7 induces CHK2 phosphorylation and interacts with ATM

**Figure 7:**
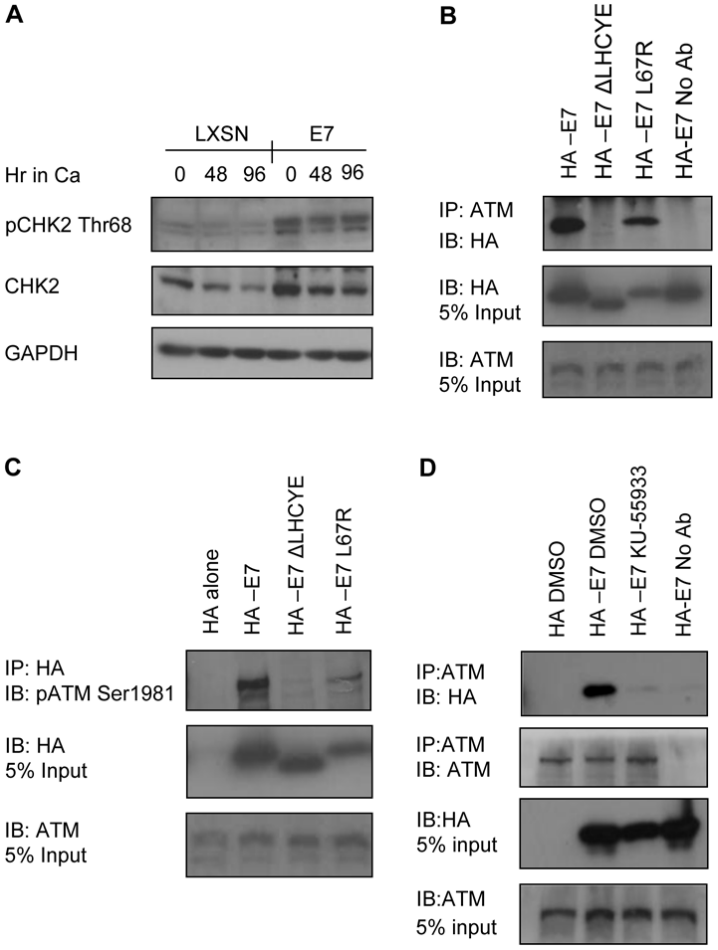
E7 induces CHK2 phosphorylation upon differentiation and interacts with the phosphorylated form of ATM. (A) Lysates were harvested from HFKs transduced with the retroviral vector pLXSN alone, or pLXSN-HPV-31 E7 at 0 hr (undifferentiated cells), as well as after differentiation in high calcium for 48 and 96 hours. Western blot analysis was performed using a phospho-specific antibody to pCHK2 Thr68, as well as an antibody to total CHK2. GAPDH served as a loading control. Ca = calcium. (B) Lysates were prepared from U2OS cells transiently transfected with vectors expressing HA, wild-type HA-31E7, HA-31E7 ΔLHCYE or HA-31E7 L67R. Immunoprecipitations were performed using an antibody to endogenous ATM, followed by Western blot analysis using an antibody to HA. (C) Immunoprecipitations were performed on lysates harvested from U2OS cells transfected with HA alone, HA-E7, HA-E7 DLHCYE or HA-E7 L67R using an antibody to HA followed by Western blot analysis to detect precipitated pATM Ser1981. (D) Lysates were prepared from transfected U20S cells treated with DMSO or 10 uM KU-55933 for 12 hours. Immunoprecipitatations were performed using an antibody to ATM, followed by Western blot analysis using an antibody to HA. IP = Immunoprecipitation, IB = Immunoblot. No Ab = No antibody (control).

To determine which viral proteins could be responsible for ATM activation, we first examined a possible role of E7 in this process, since it has been shown to de-regulate cell cycle control upon differentiation [46]. Our studies indicate that keratinocytes stably expressing HPV-31 E7 from the pLXSN retroviral vector exhibit phosphorylation of CHK2 on Thr68 in undifferentiated and differentiated keratinocytes, with little activation observed in cells expressing the pLXSN vector alone (Figure 7A). Since E7 mediates the majority of its functions through protein-protein interactions [46], we next investigated if E7 interacts with ATM. For these studies, we utilized the osteosarcoma cell line U2OS, which contains both wild-type p53 and Rb. U2OS cells were transfected with expression vectors for HA-tagged HPV-31 E7, HA-E7 lacking the LXCXE Rb binding motif (ΔLHCYE), or with an HA-E7 HDAC binding mutant (L67R). Following transfection, proteins associated with endogenous ATM were isolated by immunoprecipitation using an antibody to ATM, followed by Western blot analysis using an antibody to HA to detect precipitated E7 proteins. As shown in Figure 7B, wild-type E7 and the HDAC binding mutant were both able to co-immunoprecipitate with ATM, although the HDAC binding mutant was less efficient in doing so. In contrast, the interaction between E7 and ATM was abrogated in cells transfected with the LXCXE Rb-binding mutant, which is also defective for productive HPV replication [47]. Several proteins in addition to Rb have been shown to interact with E7 at this site [46], and it is unclear whether the binding of E7 to ATM is direct or is mediated through another protein or complex of proteins. We also performed immunoprecipitations using an antibody to the HA tag, followed by Western blot analysis for pATM Ser1981, and found that E7 is able to bind the phosphorylated form of ATM (Figure 7C). Again, the HDAC binding mutant was still able to interact with pATM, but to a lesser degree than wild-type E7. We consistently observed that the L67R mutant was expressed at lower levels than wild-type HA-E7, as well as the Rb-binding mutant, and likely accounts for the decreased binding of this mutant to ATM. To determine if ATM phosphorylation is necessary for E7 to interact with ATM, U20S cells transfected with wild-type HA tagged E7 were treated with 10 uM of the ATM inhibitor KU-55933, which inhibits the phosphorylation of ATM (Figure 1D). Endogenous ATM was precipitated, followed by Western blot analysis using an antibody to HA to examine the presence of E7 in the precipitated complexes. As shown in Figure 7D, E7 co-precipitated with ATM in cells treated with DMSO alone, but not in cells treated with the ATM inhibitor. Since total levels of ATM were not affected by treatment with the ATM inhibitor (Figure 7D), these results suggest that E7 interacts primarily with the phosphorylated form of ATM. Overall, these results indicate that E7 activates CHK2, possibly through its association with ATM, which may in turn be important for the productive phase of the viral life cycle.

## Discussion

In this study, we show that human papillomaviruses activate the ATM DNA damage response and that this is necessary for productive viral replication upon differentiation. These findings identify a primary regulatory mechanism responsible for HPV genome amplification. Our studies indicate that papillomaviruses induce phosphorylation of ATM, as well as its substrates CHK2 and BRCA1, in undifferentiated cells, but this activation has minimal effect on the long-term maintenance of HPV episomes. In differentiating cells, the phosphorylation of NBS1, well as CHK2 and BRCA1, was observed and inhibition of the ATM pathway completely blocked amplification of viral genomes. In addition, we found CHK2 activity to be required for HPV-mediated caspase activation, as well as viral genome amplification. It is possible that activation of the ATM response in differentiating cells induces an S or G2/M arrest that provides an environment conducive to productive viral replication. HPV genomes replicate bi-directionally via theta structures in basal cells, but may switch to replication by a rolling circle mechanism during amplification [48]. This switch in replication modes may also require activation of the ATM pathway specifically in differentiating cells.

HSV-1 and SV40 have been shown to recruit members of the ATM DNA damage pathway to specific sites of replication in the nucleus [49],[50],[51]. In differentiating cells undergoing productive replication of HPV genomes, we observed the formation of nuclear foci containing pATM, pCHK2 and γ-H2AX, as well as the MRN components, MRE11 and RAD50. The addition of ATM inhibitors prevented formation of these foci and blocked viral genome amplification, further implicating these DNA repair proteins as important regulators of HPV productive replication. We did not observe a complete loss in the formation of γ-H2AX foci upon treatment with the ATM inhibitor, however this may be due to the actions of other checkpoint kinases, such as ATR and DNA-PK [27]. Consistent with this idea, we observed ATM-independent phosphorylation of the ATR target CHK1 on Ser317 in undifferentiated cells, as well as in differentiated cells, although at decreased levels.

Our studies further demonstrate that the addition of a specific inhibitor to CHK2 blocks viral genome amplification. Upon activation, CHK2 phosphorylates the Cdc25a and Cdc25c phosphatases to initiate cell cycle arrest in G1/S or G2/M through their degradation or cytoplasmic sequestration, respectively [52]. Cdc25c is necessary for the activation of Cdc2/Cdk1, as well as entry into mitosis, and its inactivation plays a central role in inducing G2/M arrest [53]. Preliminary results indicate that phosphorylation of Cdc25c on Ser216 increases in HPV positive differentiating cells (Figure S4), as does inhibitory phosphorylation of Cdc2/Cdk1 on Tyr15 (Moody and Laimins, unpublished data), and this occurs in a CHK2-dependent manner. These results are consistent with ATM-dependent activation of CHK2 leading to a G2/M arrest and viral genome amplification. Recent studies suggest that HPV infected cells undergoing productive replication are arrested at G2/M rather than in S-phase and is consistent with our findings [54].

The MRN complex appears to be important for HPV amplification, as the phosphorylated forms of CHK2, NBS1 and BRCA1 were detected in differentiated HPV positive cells. For some viruses, the DNA damage response represents an obstacle that must be overcome for efficient replication. For example, the adenovirus E1b55K/E4orf6 proteins induce degradation of the MRN complex, blocking NBS1 phosphorylation and preventing concatemerization of viral genomes [15],[39]. In contrast, differentiating HPV positive cells exhibited high levels of NBS1, MRE11 and RAD50, which were maintained throughout differentiation. In addition, we observed that NBS1 phosphorylation occurs concomitantly with viral genome amplification, implicating NBS1 as a potential regulator of replication. The Rb associated transcription factors E2F1 and E2F2 have been shown to interact with MRE11 and NBS1 at both viral and cellular origins of replication [55]. Upon differentiation, E7 induces increased expression of E2F2, as well as its re-localization to nuclear foci, which is necessary for viral genome amplification [56]. Recent studies indicate that E2F2 binds to the region around the HPV-31 replication origin and increased binding was observed upon differentiation [57]. It is possible that E2F2 directs MRN components to HPV origins to ensure integrity of replication forks and promote replication in differentiating cells.

Caspase activation has been shown to be an important and novel means by which HPV proteins regulate amplification [7]. Low level caspase activation by E6 and E7 upon differentiation induces cleavage of the E1 protein, which is required for efficient viral genome amplification. Cleavage of E1 results in enhanced binding of E1 to the origin and the ability to replicate in an E2-independent manner (Moody, Archambault et al. unpublished data). In the present study, we have found that CHK2 kinase activity is necessary for caspase activation, as well as viral genome amplification in differentiating cells. These results provide a possible link between caspase cleavage and the activation of CHK2 through the binding of ATM to E7. In preliminary studies, we have found that E6 can also activate CHK2 (Moody and Laimins, unpublished data), although it is unclear whether E6 utilizes the DNA damage response to induce caspase activation in differentiating cells. Several recent studies have demonstrated a role for CHK2 in DNA damage-induced apoptosis [16],[43],[44],[45]. In response to DNA damage, CHK2 phosphorylates E2F1, resulting in its stabilization, transcriptional activation, and induction of p53-dependent or -independent apoptosis [16],[43]. E2F1 is expressed at high levels in differentiating HPV positive cells [56], and it is possible that CHK2 mediates caspase activation through E2F1. Interestingly, our studies indicate that HPV induces caspase activation only in differentiating cells, while activation of CHK2 is observed in both undifferentiated and differentiated cells. This suggests that while CHK2 activation is necessary for caspase activation, it alone is not sufficient and may require differentiation-specific factors as well as other members of the ATM pathway for this function. Several viruses have been shown to utilize DNA damage response for productive replication, and it is possible that these viruses also utilize low-level caspase activation as part of their life cycle.

Our studies indicate that in stable cell lines E7 activates CHK2, and that it forms a complex with the phosphorylated form of ATM. Deletion of the LXCXE Rb binding domain in E7 abrogated ATM binding, however binding could occur directly or indirectly through another protein. Preliminary results using Rb-deficient Saos-2 cells indicate that E7 binding to ATM does not require Rb (Moody and Laimins, unpublished data). Previous studies have shown that deletion of the LXCXE binding domain in E7 blocks HPV genome amplification [47], and in our studies this motif is important for ATM binding. This is consistent with the idea that E7's interaction with ATM may be necessary for productive replication.

In addition to E7, the HPV replication protein E1 may also contribute to the ATM response. Upon differentiation, the expression of E1 increases, contributing to enhanced viral replication [58]. Kadaja et al demonstrated that heterologous high-level expression of E1 initiates replication from integrated HPV origins multiple times in a single S-phase [59]. This suggests that E1 may disrupt normal licensing control in differentiating cells, allowing for re-replication of HPV DNA and activation of an ATM response. It is possible that multiple HPV proteins act cooperatively to activate the full ATM response.

In summary, our studies demonstrate that HPV proteins activate the ATM DNA damage response and that this is necessary for amplification of viral genomes upon differentiation. The formation of HPV replication foci in differentiating cells is dependent upon ATM activity and suggests that DNA repair proteins may participate directly in viral replication. Importantly, we have established a link between caspase activation and the DNA damage response. Caspase 3/7 consensus cleavage motifs are found at conserved locations in E1 proteins of almost all papillomavirus types, and we suspect caspase activation may be necessary for their productive replication. We believe that activation of the ATM pathway will prove to be a common mechanism utilized by HPVs to promote viral replication in differentiating cells. These observations suggest that the ATM pathway may be an effective therapeutic target to block the spread of HPV infections.

## Materials and Methods

### Cell culture

Human foreskin keratinocytes (HFKs) were derived from neonatal human foreskin epithelia and maintained in E medium containing mouse epidermal growth factor (EGF) and mitomycin-treated J2 fibroblasts as previously described [60]. Human osteosarcoma cells (U2OS) were maintained in Dulbecco's modified Eagle's medium (DMEM) containing 10% bovine serum. CIN612 is a clonal cell line that stably maintains HPV-31 episomes. CIN612 cells were maintained in E medium with EGF and J2 fibroblast feeders. Before harvesting DNA or protein, fibroblast feeders were removed by treatment with phosphate-buffered saline (PBS) containing 0.1% of .5 M EDTA for two minutes, followed by two washes in PBS. Creation of HFK cell lines containing retroviral constructs has been previously described [47].

### Plasmids and chemicals

The pBR322min-HPV31 plasmid has been described [61]. The HA-tagged E7 proteins were previously described [47] and are as follows: HA-E7 ΔLHCYE contains an in-frame deletion of the Rb binding domain and HA-E7 L67R contains a point mutation in the HDAC binding site, converting a leucine to an arginine. KU-55933 and the CHK2 inhibitor II were obtained from Calbiochem.

### Stable transfection of HFKs

Transfection of HFKs and selection for cells stably maintaining HPV-31 genomes were performed as described previously [62]. Briefly, HPV-31 genomes were released from the pBR322 plasmid by digestion with HindIII. Viral genomes were then unimolecularly ligated with T4 DNA ligase (New England Biolabs) and precipitated with isopropyl alcohol. HFKs were co-transfected with 1 ug of religated genomes and 1 ug pSV2-Neo using FuGene6 according to the manufacturer's protocol (Roche). Selection was carried out for eight days in the presence of G418 (Sigma). After selection was complete, pooled populations were expanded for further analysis.

### Calcium induced differentiation

Differentiation in high calcium was performed as described previously [7]. Briefly, upon reaching 90% confluency, HPV-positive cells and normal HFKs were cultured in keratinocyte basal medium (KBM) with supplements (Invitrogen) for 24 hours. Cells were then switched to KBM (without supplements) containing 1.5 mM calcium chloride, and where indicated were cultured with either DMSO, 5 or 10 uM KU-55933, or 5 uM of the CHK2 inhibitor. At 48 and 96 hours, DNA was harvested from one half of the cells, and protein was harvested from the other half. Viral genome amplification was then measured by Southern blot analysis to examine the episomal (supercoiled) form of DNA to ensure that the productive phase of the viral life cycle was activated. Western blot analysis was performed to analyze the expression of differentiation-specific proteins.

### Western blot and Southern blot analyses

Whole cell extracts were harvested in RIPA lysis buffer and quantified using the Bio-Rad protein assay. Western blot analysis was performed as described [47]. Equal amounts of protein were electrophoresed on SDS-polyacrylamide gels and subsequently transferred to polyvinylidene difluoride membrane (Immobilon-P; Millipore). Primary antibodies were as follows: phospho-ATM Ser1981 was purchased from R&D Systems. Phospho-CHK2 Thr68, CHK2, BRCA1, K10 and Involucrin were purchased from Santa Cruz. ATM was purchased from Calbiochem. Phospho-CHK1 Ser317, CHK1, phospho-CHK2 Thr68, phospho-BRCA1 Ser1524, Cleaved Caspase-7, and Caspase-7 were purchased from Cell Signaling. Phospho-NBS1 Ser343, MRE11 and RAD50 were purchased from Genetex. NBS1 was purchased from Novus Biologicals. Secondary antibodies included horseradish peroxidase-linked anti-rabbit (Cell Signaling Technologies) and horseradish peroxidase-linked anti-mouse (Santa Cruz). DNA isolation and Southern blot analysis were performed as described [63].

### HA-E7 protein expression and immunoprecipitation

U20S cells were transfected at 30% confluency with one microgram of wild-type or mutant HA-tagged HPV-31 E7 proteins using FuGene6, according to the manufacturer's instructions (Roche). After 48 hours, lysates were harvested as previously described [47]. Immunoprecipitations were performed using one milligram of protein lysate. The samples were pre-cleared for 1 hour with 40 µl of protein G agarose (Roche) at 4°C, then incubated overnight with either a mouse anti-HA antibody (Santa Cruz) or rabbit anti-ATM antibody (Calbiochem). Protein complexes were then precipitated using 50 µl of protein G agarose for 4 hours at 4°C. Immunoprecipitated complexes were then washed three times with lysis buffer, and subsequently analyzed by Western blot analysis using either mouse anti-HA, mouse anti-pATM Ser1981 (Rockland), or rabbit anti-ATM antibodies.

### Immunofluorescence and immunohistochemistry

HPV positive cells and normal HFKs were grown on coverslips and induced to differentiate in high calcium in the presence of DMSO or 10 uM KU-55933. At time 0 (undifferentiated) and 48 hr post-calcium induced differentiation, the cells were washed three times in cold phosphate buffered saline (PBS), fixed in 4% paraformaldehyde in PBS for 15 minutes, then permeabilized in 1%Triton X-100-PBS for 10 minutes. Cells were blocked with PBS containing 10% bovine serum albumin (BSA) for one hour at room temperature. Primary antibodies were diluted in PBS containing 10% BSA and incubated on coverslips overnight at 4°C. The samples were then washed in PBS and stained with fluoroscein isothiocyanate (FITC)-conjugated anti-rabbit antibody (1∶50 dilution) (Zymed) or AlexaFluor 568 anti-mouse antibody (Invitrogen) (1∶400 dilution) for one hour at room temperature. Primary antibody dilutions for mouse anti-phospho-ATM Ser1981 (Rockland), anti phospho-H2AX Ser139 (γ-H2AX) (Upstate) were 1∶400 and 1∶500, respectively. Rabbit anti-phospho-CHK2 Thr68 (Cell Signaling) and anti-phospho-H2AX Ser139 (γ-H2AX) (Cell Signaling) were diluted 1∶50. Mouse anti-MRE11 and mouse anti-Rad50 were diluted 1∶200. Mouse anti-keratin 10 (K10) (Santa Cruz) was diluted 1∶100. Sections from normal keratinocyte raft cultures or HPV-31 transfected keratinocyte raft cultures were examined by immunofluorescence as described previously [63]. Cross sections of rafts were stained using a 1∶50 dilution of anti-phospho-CHK2 Thr68 (Cell Signaling), 1∶200 of anti-pATM Ser1981, or MRE11, 1∶500 of γ-H2AX Ser139 (Millipore), and 1∶100 of anti-K10 (Santa Cruz). Cellular DNA was counterstained with DAPI, and the coverslips or slides were mounted in Vectashield (Vector Laboratories). Confocal images were acquired by a UV LSM 510 confocal laser-scanning microscope (Zeiss). To quantify number of cells containing DNA repair foci, at least 100 cells were counted for three independent experiments. The average number of cells containing foci is indicated, along with the standard deviation.

### Fluorescent in situ hybridization (FISH)

HPV-31 genomic DNA probes for FISH were prepared by nick translation of plasmid genomic DNAs using the BioNick labeling system according to the manufacturer's instructions (Invitrogen). Viral DNA was detected by tyramide fluorescent in situ hybridization as previously described [64]. Briefly, 1×10^6^ undifferentiated HFK-31 cells, or HFK-31 cells differentiated in high calcium for 48 hr were spread on Superfrost Plus slides (Fisher) and allowed to air-dry. Cells were fixed with 4% paraformaldehyde at room temperature followed by permeabilization in 1× PBS, 0.5% Triton X-100 for 10 min. The slides were treated with 100 ug/ml RNase A in 2× SSC for 1 hour at 37°C. Subsequently, the slides were washed three times with 2× SSC, then dehydrated for 2 min each in 70% EtOH, 85% EtOH and 100% EtOH at room temperature. Slides were then denatured in 70% formamide-2× SSC at 74°C for 2 minutes, followed by dehydration for 2 min each in 70% EtOH (−20°C), 85% EtOH and 100% EtOH at room temperature. The probe was denatured at 74°C for 10 minutes, and then 10 ul of probe was hybridized overnight to the denatured slide at 37°C. After overnight incubation, the slides were washed multiple times, and tyramide-enhanced fluorescence was carried out according to the manufacturer's instructions (Perkinelmer). The cellular DNA was counterstained with DAPI, and the slides were mounted in Vectashield. Images were collected using a UV LSM 510 confocal laser-scanning microscope (Zeiss).

## Supporting Information

Figure S1 MRN components are localized to nuclear foci in HPV positive cells. HFK-31 cells, as well as normal HFKs were harvested, fixed and permeabilized at either time 0 (undifferentiated cells) or after 48 hr of calcium-induced differentiation. Samples were stained with antibodies to either MRE11 or RAD50 and analyzed by confocal fluorescence microscopy. Cellular DNA was counterstained with DAPI and is shown as merged with the indicated antibodies.
(5.56 MB PDF)

Figure S2 DNA repair proteins exhibit a nuclear staining pattern in raft cultures of HPV positive cells. Immunohistochemistry was performed on cross sections of organotypic raft cultures generated from HFK-31 cells, as well as normal HFKs using antibodies to (A) pATM Ser1981, (B) γ-H2AX, (C) MRE11, or (D) K10. Cellular DNA was counterstained with DAPI. Images were captured using confocal fluorescence microscopy. pATM is found in the basal and suprabasal cells in HFK-31 cells but only background staining is observed in HFKs. MRE11 is distributed throughout all epithelial layers for rafts generated from HFK-31 cells, as well as normal HFKs. γ-H2AX is found at high levels in all layers of HFK-31 rafts, and at reduced levels in normal HFK rafts.
(2.77 MB PDF)

Figure S3 Southern analysis of HPV-31 cells treated with inhibitors of ATM and CHK2. DNA was harvested from undifferentiated CIN612 cells, as well as from cells induced to differentiate for 48 and 96 hr in high calcium in the presence of DMSO, 10 uM KU-55933 or 5 uM of the CHK2 inhibitor (CHK2i). Total DNA was digested with either Xho1, which does not cut the HPV genome (uncut), or with HindIII, which linearizes the genome (cut). Southern blot analysis was performed to analyze viral genome amplification. The four forms of HVP-31 DNA found in this analysis are labeled. Standards of HPV genome copies per cell are indicated to the left of the gel. Ca = calcium.
(0.80 MB PDF)

Figure S4 Analysis of the efficacy and specificity of the CHK2 inhibitor. Lysates were harvested from undifferentiated CIN612 cells, as well as after differentiation in high calcium for 48 and 96 hr in the presence of DMSO or 5 uM CHK2i. Western blot analysis was performed using an antibody to the CHK2 substrate pCdc25c Ser216, or to pCHK2 Thr68 or total CHK2. GAPDH served as a loading control. Ca = calcium.
(3.75 MB PDF)
