# Supplementary figures and images for: Human Papillomaviruses Activate the ATM DNA Damage Pathway for Viral Genome Amplification upon Differentiation
Source: PLoS Pathog. 2009 Oct 2;5(10):e1000605. doi: 10.1371/journal.ppat.1000605 (PMC2745661; doi:10.1371/journal.ppat.1000605)

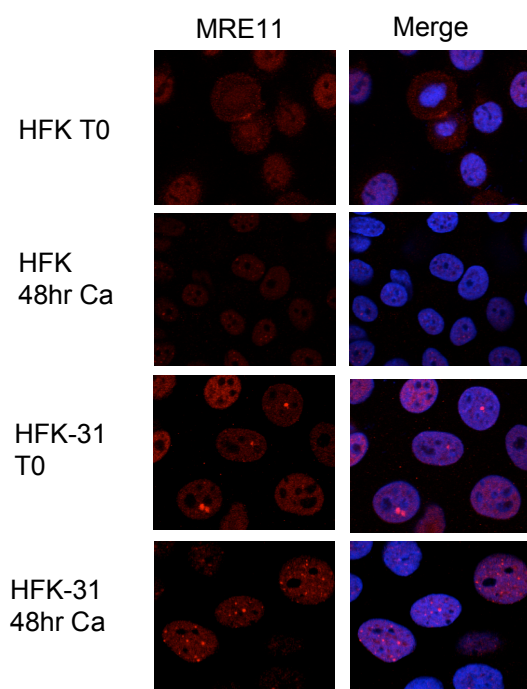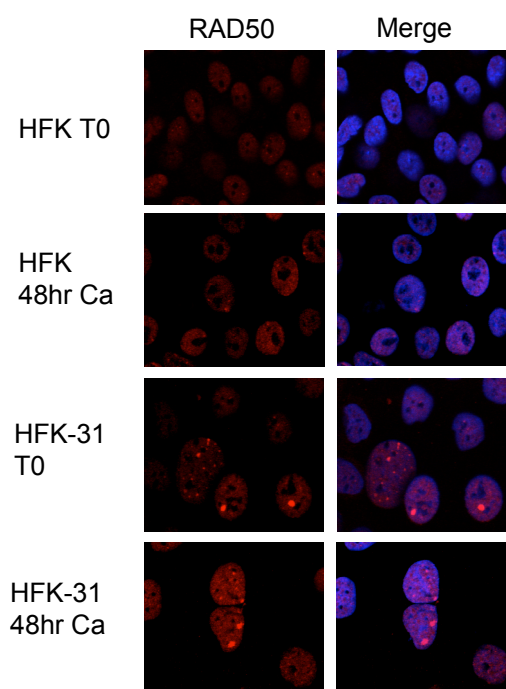

Supplement: Figure S1 — MRN components are localized to nuclear foci in HPV positive cells. HFK-31 cells, as well as normal HFKs were harvested, fixed and permeabilized at either time 0 (undifferentiated cells) or after 48 hr of calcium-induced differentiation. Samples were stained with antibodies to either MRE11 or RAD50 and analyzed by confocal fluorescence microscopy. Cellular DNA was counterstained with DAPI and is shown as merged with the indicated antibodies. (5.56 MB PDF) [file ppat.1000605.s001.pdf]

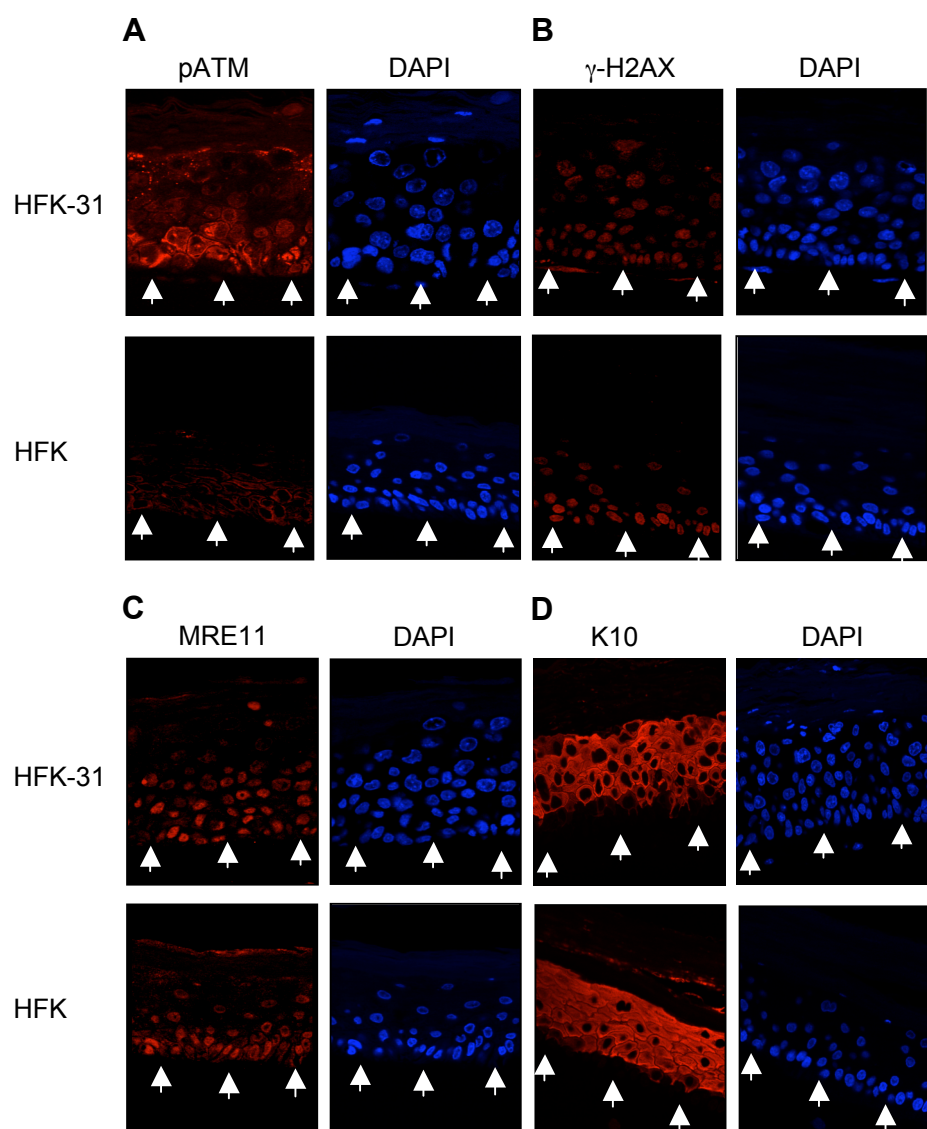

Supplement: Figure S2 — DNA repair proteins exhibit a nuclear staining pattern in raft cultures of HPV positive cells. Immunohistochemistry was performed on cross sections of organotypic raft cultures generated from HFK-31 cells, as well as normal HFKs using antibodies to (A) pATM Ser1981, (B) γ-H2AX, (C) MRE11, or (D) K10. Cellular DNA was counterstained with DAPI. Images were captured using confocal fluorescence microscopy. pATM is found in the basal and suprabasal cells in HFK-31 cells but only background staining is observed in HFKs. MRE11 is distributed throughout all epithelial layers for rafts generated from HFK-31 cells, as well as normal HFKs. γ-H2AX is found at high levels in all layers of HFK-31 rafts, and at reduced levels in normal HFK rafts. (2.77 MB PDF) [file ppat.1000605.s002.pdf]

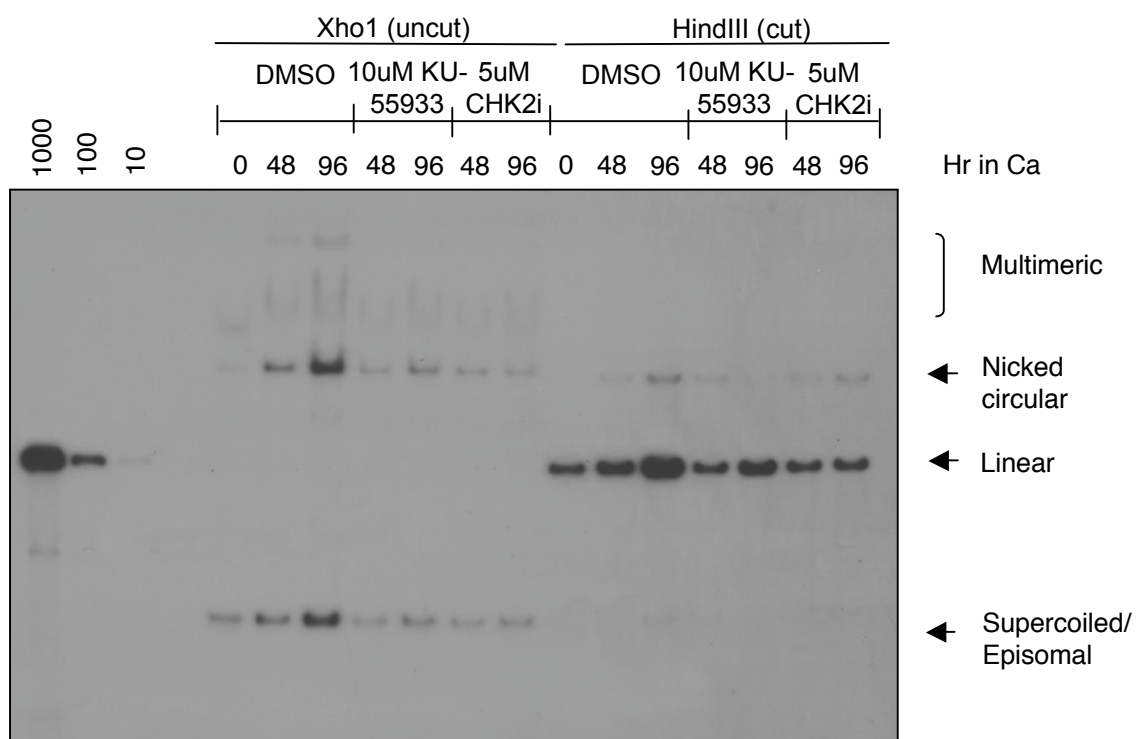

Supplement: Figure S3 — Southern analysis of HPV-31 cells treated with inhibitors of ATM and CHK2. DNA was harvested from undifferentiated CIN612 cells, as well as from cells induced to differentiate for 48 and 96 hr in high calcium in the presence of DMSO, 10 uM KU-55933 or 5 uM of the CHK2 inhibitor (CHK2i). Total DNA was digested with either Xho1, which does not cut the HPV genome (uncut), or with HindIII, which linearizes the genome (cut). Southern blot analysis was performed to analyze viral genome amplification. The four forms of HVP-31 DNA found in this analysis are labeled. Standards of HPV genome copies per cell are indicated to the left of the gel. Ca = calcium. (0.80 MB PDF) [file ppat.1000605.s003.pdf]

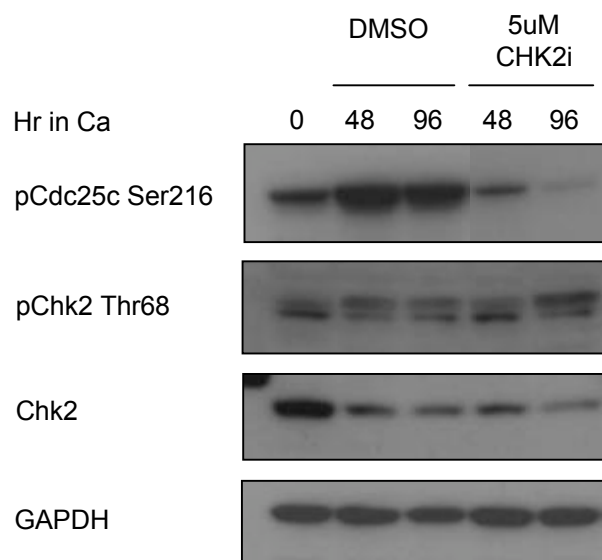

Supplement: Figure S4 — Analysis of the efficacy and specificity of the CHK2 inhibitor. Lysates were harvested from undifferentiated CIN612 cells, as well as after differentiation in high calcium for 48 and 96 hr in the presence of DMSO or 5 uM CHK2i. Western blot analysis was performed using an antibody to the CHK2 substrate pCdc25c Ser216, or to pCHK2 Thr68 or total CHK2. GAPDH served as a loading control. Ca = calcium. (3.75 MB PDF) [file ppat.1000605.s004.pdf]
